# Host-parasite interaction explains variation in the prevalence of avian haemosporidians at the community level

**DOI:** 10.1371/journal.pone.0205624

**Published:** 2019-03-06

**Authors:** Luz Garcia-Longoria, Alfonso Marzal, Florentino de Lope, Laszlo Garamszegi

**Affiliations:** 1 Departamento de Anatomía, Biología Celular y Zoología, Universidad de Extremadura, Badajoz (Spain); 2 Molecular Ecology and Evolution Lab, Department of Biology, Lund University, Sölvegatan 37, Lund, Sweden; 3 Department of Evolutionary Ecology, Estación Biológica de Doñana-CSIC, Seville, Spain; 4 MTA-ELTE, Theoretical Biology and Evolutionary Ecology Research Group, Department of Plant Systematics, Ecology and Theoretical Biology, Eötvös Loránd University, Budapest, Hungary; 5 Behavioural Ecology Group, Department of Systematic Zoology and Ecology, Eötvös Loránd University, Budapest, Hungary; Universidade Federal de Minas Gerais, BRAZIL

## Abstract

Parasites are a selective force that shape host community structure and dynamics, but host communities can also influence parasitism. Understanding the dual nature from host-parasite interactions can be facilitated by quantifying the variation in parasite prevalence among host species and then comparing that variation to other ecological factors that are known to also shape host communities. Avian haemosporidian parasites (e.g. *Plasmodium* and *Haemoproteus*) are abundant and widespread representing an excellent model for the study of host-parasite interactions. Several geographic and environmental factors have been suggested to determine prevalence of avian haemosporidians in bird communities. However, it remains unknown whether host and parasite traits, represented by phylogenetic distances among species and degree of specialization in host-parasite relationships, can influence infection status. The aims of this study were to analyze factors affecting infection status in a bird community and to test whether the degree of parasite specialization on their hosts is determined by host traits. Our statistical analyses suggest that infection status is mainly determined by the interaction between host species and parasite lineages where tolerance and/or susceptibility to parasites plays an essential role. Additionally, we found that although some of the parasite lineages infected a low number of bird individuals, the species they infected were distantly related and therefore the parasites themselves should not be considered typical host specialists. Infection status was higher for generalist than for specialist parasites in some, but not all, host species. These results suggest that detected prevalence in a species mainly results from the interaction between host immune defences and parasite exploitation strategies wherein the result of an association between particular parasite lineages and particular host species is idiosyncratic.

## Introduction

Parasites have been suggested as a selective force since they might shape host community dynamics [1], alter interspecific competition and influence energy flow [2]. An essential trait in studies of host-parasite interactions is prevalence (i.e. the proportion of individuals infected by a parasite or pathogen in a population at one point in time [3]). The importance of prevalence is reflected in the amount of studies showing how major ecological factors shaped its intra- and interspecific variation [4,5]. In bird communities, geographic areas, environmental issues or host species/population have been suggested as factors determining prevalence [6]. However, more studies are still needed in order to understand the main factors affecting parasite prevalence at the community level.

Inferences about host-parasite dynamics based on prevalence may sometimes be difficult, because by definition prevalence combines all host individuals within the same species by a single metric. This approach inherently disregards within-species variance in susceptibility to parasitism. However, considerable among-individual differences may arise due to the fact that different host individuals are in different age or in condition or under the influence of different environmental factors, which all affect the probability of being found infected by a particular parasite [7–9]. Accordingly, if a population sample is composed of individuals with different infection status, detected prevalence can vary within a species depending on the individual composition of the sample. As a result, prevalence can be remarkably different in the same species if it is sampled under different environmental conditions [10,11]. Therefore, to understand how parasite prevalence is shaped at the community level, it is also crucial to account for among-individual differences in infection status that helps elucidate mechanisms that act at the within-species and the between-species level in the host.

Avian *Plasmodium* and *Haemoproteus* spp. represent a well-studied host-parasite system. These parasites are vector-transmitted organisms that can cause host mortality or morbidity during the acute phase of infection impacting the life histories of their hosts [12]. Their life-cycle is complex, involving sexual stages in their dipteran vectors and asexual stages in their vertebrate hosts [12]. Prevalence and infection status of these parasites may be affected by different factors concerning the vector, hosts and the parasites themselves [12]. Some authors have focused their attention on the possibility that host traits might determine prevalence of these parasites [13–15]. For instance, blood parasite susceptibility of both bird individual [16] and bird species [17,18] have been recently suggested as factors affecting prevalence. In this sense, some bird species may have developed species-specific tolerance and resistance mechanisms [19] underlying the importance of bird species as units in prevalence studies. However, given that individuals within the same species can also depict remarkable variation in terms of susceptibility to parasitism, it would also be essential to account for this variation in interspecific studies of prevalence.

In addition to characteristics of hosts, characteristics of parasites themselves can affect their prevalence in wild communities. Haemosporidians parasites present high plasticity and versatility reflected in the number of parasite lineages found among bird species. Thus, within haemosporidian parasites there are more than 3000 parasite lineages [20] infecting more that 1500 bird species. Each of these lineages may have different virulence [21], which could result in an array of different negative effects within the hosts. The plasticity that malaria lineages may have [22,23] allow these parasites to fully exploit hosts making them able to respond to changes in the physiological state of the host or the environment [24]. Thus, prevalence displayed by parasite lineage in a determined host species or individual is simply the interaction between host immune defences (susceptibility or tolerance) and parasite strategy (virulence). Additionally, malaria parasites must find the correct host in order to complete its life cycle and achieve a high transmission rate [3,25]. In this sense, two main hypotheses have been suggested to explain the evolutionary mechanisms driving the positive abundance-occupancy relationship (AOR) in parasites. On the one hand, the trade-off hypothesis (TOH) suggests that parasites exploiting many host species will achieve lower mean abundance in these hosts than more host-specific parasites because of the presumably higher costs of adaptations against multiple defence systems [26]. On the other hand, the resource breadth hypothesis (RBH) predicts that the same attributes that enable species to exploit a variety of hosts allow them to attain higher densities in these hosts [27]. Thus, a positive correlation between distribution and local abundance is expected, where generalist parasites will attain higher prevalence than more host-specific parasites. These two hypotheses have been broadly discussed and specialist parasites are supposed to display higher prevalence and parasitemia (TOH) on their avian hosts [28] while generalist parasite can occasionally cause significant mortalities in native birds. However, and despite the large number of studies focused on haemosporidian parasites, there are a scarce number of them dealing with factors affecting prevalence in wild communities from the perspectives of both the host and parasites.

*Plasmodium* and *Haemoproteus* spp. might be detected in several host species that can be distantly or closely related to each other [29–31]. Mechanisms allowing the parasite to switch host is a central issue nowadays in avian malaria studies [32,33]. Host switching mechanism is a strategy that malaria parasites may use, in order to infect larger number of bird species [33,34], whereby closely related parasite lineages may be detected in bird species with similar immune defences [35–37]. Thus, parasite lineages infecting closely related bird species may exhibit the same exploitation strategy to avoid host defences [38]. Conversely, more generalist parasites would be able to infect a broad range of bird species that might be distantly related [39]. Analysing phylogenetic distances between bird species sharing the same parasite lineages becomes essential in community studies in order to determine parasite strategies and how specialist and generalist parasites exploit different host species. However, little is know about how haemosporidian parasite lineages infect bird host species depending on the host phylogenetic distance.

The fact that haemosporidian parasites need a vector for completing their cycle life extends the number of elements affecting prevalence of these parasites. Although some studies have shown limited or no relationship between climate and prevalence [40], some have shown that vector populations might increase their number due to climate change and, therefore, vector-borne infectious diseases may increase every year [41]. Vector availability might also change among seasons, thus, during summer the amount of vectors in the wild increase [42] raising the probabilities to become infected [43]. During this season, the probability of host individuals of becoming infected increases significantly not only because of vector availability but also because of the secretion of sexual hormones that may alter immune system and, therefore, facilitate the entrance of blood parasite in the vertebrate host [44]. These studies emphasize the importance of including both year and season of sampling for studies focusing on host-parasite dynamics of haemosporidians when analysing communities.

The main aim of this study is to analyse factors that potentially affect infection status by haemosporidian parasites in a wild community of birds. We will work at the individual level and examine if the incidence of infection by different haemosporidian parasites is influenced by aspects at the individual level (identity and seasonal status), species-specific effects (i.e. effects due to species identity and phylogeny) and parasite-specific effects (i.e. effects due to lineage identity and phylogeny). Furthermore, we will also compare the phylogenetic distances among bird species infected with the same parasite lineage in order to determine whether the same parasite lineage infects host species that are phylogenetically related.

## Materials and methods

### Data collection and database

The study was carried out from February to October during a 9-year period (2002–2010) in the surroundings of Badajoz (SW Spain) (38°53’00”N 06°58’00”W). Birds were collected always during the sunrise and during the early hours of the morning. Using a mist-net system, we captured a total of 815 adult individuals from 21 bird species belonging to nine different families. We obtained one microcapillary of blood (70μl) from the brachial vein of each individual and stored it in 500 μl of 96% ethanol until analysis. Ethanol (96–100%) is an excellent killing agent and preservative for tissues [45,46], and it has been widely used to store avian blood samples for molecular analyses [47,48]. Our sampling did not recapture any birds, meaning that the present study does not include any recapture. Upon capture we recorded the following variables: calendar date, year and if the bird species was breeding or not.

Methods were approved by Institutional Commision of Bioethics of University of Extremadura (CBUE 49/2011). The field studies did not involve endangered or protected species. All sampling procedures and/or experimental manipulations were reviewed or specifically approved as part of obtaining the field permit by FDL (expert bird ringer 500001 SEO/Birdlife; group: Hirundo).

### Prevalence and genetic detection of parasite lineages

DNA from the avian blood samples was extracted in the laboratory using a standard chloroform ⁄ isoamylalcohol method [49]. Diluted genomic DNA (25 ng/μL) was used as a template in a polymerase chain reaction (PCR) assay for detection of the parasites using the nested-PCR protocols described [50]. The amplification was evaluated by running 2.5 μL of the final PCR on a 2% agarose gel. All PCR experiments contained one negative control for every eight samples. In the very few cases of negative controls showing signs of amplification (never more than faint bands in agarose gels), the whole PCR-batch was run again to make sure that all positives were true. All samples with positive amplification were sequenced directly using procedures described in [51]. The obtained sequences of 478 bp of the cyt b were edited, aligned and compared in a sequence identity matrix using the program BioEdit [52]. Parasites with sequences differing by one nucleotide substitution were considered to represent evolutionary independent lineages [53]. Five new sequences were deposited in GenBank under the accession numbers JQ749720 –JQ749724. We use the software MEGA7 [54] for creating pairwise distance matrix.

### Phylogenetic relatedness of host and parasites

For our statistical models (see below) we created two different phylogenetic trees (supporting information S1 Fig) to control for the common descent of different parties: one for the host of bird species (model 1) and one for the parasite lineages (model 2). The first one relied on the 1000 trees generated by the birdtree.org website [55], from which a consensus tree was created for the sampled 21 species by using Geneious v5.4 [56]. The second tree relied on the sequences of the parasite lineages [20] identified in this study. Then, we created a phylogenetic tree using MrBayes 3.1 [57]. We used jModelTest 0.1 [58] in order to determine which parasite tree offered the best fit to our data. The burn-in was identified through Tracer 1.2.2 [59]. We sampled 10 million generations at intervals of 1000. Finally we analysed the files generated by Bayesian MCMC runs by MrBayes in Tracer with the objective of confirm whether the parasite tree generated was the most adequate to our analyses. These phylogenetic trees were entered in the subsequent statistical models sequentially to evaluate the same list of predictors (first, we run the model using the bird phylogenetic tree (model 1) and then we run the same model but we used the parasite phylogenetic tree (model 2)).

### Statistical analyses

Given that our parasite screening method allowed us to detect infection status (yes or no) at the individual level for each parasite lineage screened, we regarded these screen results as multiple observations within the same individuals. Therefore, for our statistical modelling we focused on these observations as the units of analyses, while incorporating higher-level hierarchical effects due to host individuals in the statistical model. Accordingly, we applied a generalized mixed models (GLMM) to partition different variance components and to assess the effect of predictors on the binary state variable describing if a particular individual was infected by a particular parasite lineage or not. Accordingly, our general model incorporated the following fixed effects: date (reflecting environmental effects), breeding season (yes or no); and the following random effects: identity of individuals, identity of host species and identity of parasite lineage, the interaction between the latter two (the combination of bird species and parasite lineage by simply using the interaction between the organism names), year of sampling, and the phylogeny of either the host (model 1) or the parasite (model 2). The structure of our focal model defining the probability of a given individual of a host species being infected by a given parasite lineage in a given year (*p*_*isjk*_) can be described by this general formula:

$$Y_{isjk}∼Bern(p_{isjk})$$

$${logit(p}_{isjk})=\beta_{0}+I_{i[s,k]}+S_{s}+L_{j}{+(SL)}_{sj}+{a_{s}+K_{k}+\beta }_{1}x_{1_{i}}{+\beta }_{2}x_{2_{i}}$$

where *Y*_*isjk*_ is the binary infection status of individual *i* of host species *s* for parasite lineage *j* in year *k* (it is 1 if it is infected by parasite *j*, and 0 otherwise), *Bern*() denotes the Bernoulli distribution, *β*_0_ is the overall community-specific intercept, *I*_*i*[*s*,*k*]_ is the individual effect nested within host species *s* and year *k*, *S*_*s*_ is the host-species-specific effect, *L*_*j*_ is the parasite-lineage-specific effect, (*SL*)_*sj*_ is the interactive effect of the latter two, *a*_*s*_ is the effect attributed to host phylogeny in model 1 (can be replaced by *a*_*j*_, which then incorporates effects due to parasite phylogeny for model 2), and *K*_*k*_ is the effect for year *k* when individual *i* was sampled. These constitute the dispersion (random) part of the model. In the mean (fixed) part of the model, *x* variables are the main predictors and *β* are their corresponding regression parameters. The fixed variables we considered were the date of sampling and the breeding status of individuals (breeding or not). These two factors were included as fixed factors because they may affect infection status due to mosquito abundance/activity and susceptibility of hosts during the demanding chick-feeding period [12]. In order to avoid possible multicollinearity between the two fixed factors, the variable day was standardized [60]. To complete the above model, the following assumptions should be hold:

$$I_{i[s,k]}∼N(0,\sigma_{I}^{2})$$

$$S_{s}∼N(0,\sigma_{S}^{2})$$

$$L_{j}∼N(0,\sigma_{L}^{2})$$

$$\left({SL)}_{sj}∼N(0,\sigma_{SL}^{2}\right)$$

$$K_{k}∼N(0,\sigma_{K}^{2})$$

$$a_{s}∼N(0,\sigma_{a}^{2}A_{s})$$

where *N*() denotes the normal distribution, *σ*^2^-s are the corresponding variance components (among-individual, among-species, among-lineage, among the combination of parasite and hosts, among-year and phylogenetic variance, respectively), and *A*_*s*_ is the phylogenetic relatedness matrix as defined by the host phylogeny (can be replaced by *A*_*j*_, if *a*_*j*_ is modeled along model 2).

To fit the above model, we used the Bayesian framework for GLMM incorporating Markov chain Monte Carlo (MCMC) estimation available in the package ‘MCMCglmm’ [61]. Because dependent variable (a given parasite lineage detected or not in a given individual of a given species) is a binary state variable, we adopted the “categorical” family of the available error distribution. Year of sampling, parasite lineage, bird individual, host species and phylogenetic relationships (parasite or host) were used as random factors. Additionally, we included the interaction between host species and parasite lineage as a random factors. Plots were made with the R package ggplot2 v. 2.1.0 [62]. All the statistical analyses were carried out with the program R v.1.1.383 [63].

The model reports the mean of the posterior density distribution of model parameters with 95% credible intervals (CI) for independent variables, which indicate the precision of an estimate. Since the model provides parameter estimates (regression slopes) on the logit scale, we back transformed the results for the fixed effects by using the formula e ^mean^ / (1+ e ^mean^) in order to assess the determinants of the probability of infection on the level of host individuals. In order to explain the effect of random factors, we calculated the marginal parameter modes using kernel density estimation [61] to partition the variance in the dependent variable (and variance components) on logit scale that is explained by each random factor with 95% credible intervals (CI).

We used uninformative priors with a low degree of belief in all parameters due to the scarce prior knowledge about the effect of all these variables on our model, according to the standards suggested by Hadfiled [61]. The model was run for 180000 iterations preceded by a burn-in of 30000 iterations, and sampling every 100 iterations to avoid autocorrelation. Models were run several times in order to avoid convergence.

We examined phylogenetic relatedness of bird species infected by the same lineages, and determined if species with non-zero prevalence are more closely related to each other than could be expected by chance. For this analyses, therefore, we worked on species-level prevalences. The significance of this metric was tested by comparison to a null distribution derived from 999 random permutations among the tips of the phylogenetic tree followed by calculation of the mean pairwise distance (MPD). Calculations were carried out with the ses.mpd function of the R package Picante [64]. That analysis provides different values that may determine the MPD. The value called “MPDobs” defines the observed mean pair-wise phylogenetic distance between all species pairs infected with the parasite. The mean and standard deviation of MPD in the null distribution were obtained by randomization of species in the phylogenetic distance matrix (taxa.labels method in Picante). The value called “Z” is calculated by following this formula (MPDobs–mean MPD of the null distribution)/SD of the null distribution. Negative values of Z indicate greater phylogenetic homogeneity (clustering). P is the probability of drawing an MPD from the null distribution at least as extreme as MPDobs, based on 999 randomizations.

## Results

### Total prevalence and lineages detected

We determined the presence of 26 parasite lineages belonging to the genera *Haemoproteus* (N = 13) and *Plasmodium* (N = 13) in the analysed host species (Table 1). We found that 63.80% of total individuals and 81% of species were infected by haemosporidian parasites. We detected five new parasite lineages that had not been previously described (Table 2 and S1 Table).

**Table 1:** Prevalence and genetic identity of haemosporidian parasite lineages in Passeriform host species analyzed in this study and parasite lineage found in each host species.

Species	Migratory behaviour	Total prevalence (%) (N infected / N tested)	*Haemoproteus* lineages and specific prevalence (%)	*Plasmodium* lineages and specific prevalence (%)
*Aegithalos caudatus*	Sedentary	0 (0 / 4)		
*Certhia brachydactyla*	Sedentary	0 (0 / 8)		
*Carduelis cannabina*	Migratory	20 (1 / 5)		SGS1 (20%)
*Carduelis carduelis*	Partial migratory	18.75 (3 / 16)	CARDUEL1 (12,5%), CARDUEL2 (6 (6,25%)	
*Serinus serinus*	Sedentary	6.67 (1 / 15)	CCF2 (6.67%)	
*Carduelis chloris*	Sedentary	16.67 (1 / 6)	CARDUEL3 (16.67%)	
*Fringilla coelebs*	Sedentary	10.0 (1 / 10)		SGS1 (10.0%)
*Delichon urbicum*	Migratory	68.55 (266 / 388)	DELURB1 (17%), DELURB2 (18%), DELURB3 (0.25)	DELURB5 (2%),RTSR1 (0.02%), GRW4 (1%), GRW2 (0.2%), DURB6 (0.7%), SGS1 (14%)
*Hirundo rustica*	Migratory	17.35 (30 / 168)	DELURB2 (0.05%)	DELURB5(0.05%), GRW9 (17%)
*Riparia riparia*	Migratory	14.28 (4 / 28)	CCF2 (14.28%)	
*Cyanistes caeruleus*	Sedentary	33.33 (2 / 6)		SGS1 (33.33%)
*Parus major*	Sedentary	25 (2 / 8)	HIPOL1 (12.5%)	SGS1 (12.5%)
*Passer domesticus*	Sedentary	64.57 (177 / 275)	PADOM5 (17%), PADOM22 (0.7%), PAHIS1 (0.7%)	PADOM8 (0.3%), PADOM1 (2%), COLL1 (0.3%), PADOM2 (0.1%), GRW11 (0.6%), SGS1 (0.6%),
*Passer hispaniolensis*	Sedentary	67.74 (21 / 31)	PADOM5 (4%)	SGS1 (40%), PADOM1 (13%)
*Sturnus unicolor*	Sedentary	0 (0 / 5)		
*Phylloscopus collybita*	Migratory	7.14 (1 / 14)	HIPOL1 (7.14%)	
*Sylvia atricapilla*	Sedentary	28.57 (2 / 7)		SGS1 (28.57%)
*Sylvia melanocephala*	Sedentary	12.12 (4 / 33)	SYMEL1 (3.03%)	SGS1 (9.09%)
*Erithacus rubecula*	Sedentary	16.67 (1 / 6)	ROBIN1 (16.67%)	
*Luscinia megarhynchos*	Migratory	0 (0 / 15)		
*Turdus merula*	Sedentary	30 (3 / 10)		TURDUS3 (10.0%), SYAT05 (20%)
		Mean = 63.80 (520 /815)		

**Table 2:** Cytochrome b parasite lineage, tentative parasite species names, GenBank accession numbers and the number of host species infected by parasite lineage in the current study.

Parasite lineage	Parasite species	GenBank acc. Number	N host species infected	MalAvi N host species
COLL 1	*Plasmodium sp*.	AY831747	1	13
DELURB1	*Haemoproteus hirundinis*	EU154343	1	3
DELURB2	*Haemoproteus sp*.	EU154344	2	2
DELURB3	*Haemoproteus sp*.	EU154345	1	1
DELURB5	*Plasmodium sp*.	EU154347	2	9
DURB6	*Plasmodium sp*.	EU219392	1	1
GRW11	*Plasmodium relictum*	AY831748	1	44
GRW2	*Plasmodium ashfordi*	AF254962	1	15
GRW4	*Plasmodium relictum*	AF254975	1	79
GRW9	*Plasmodium sp*.	EU810681	2	76
HIPOL1	*Haemoproteus sp*.	DQ000324	2	4
ROBIN1	*Haemoproteus attenautus*	AY393807	1	4
CCF2	*Haemoproteus sp*.	AF495551	2	6
**CARDUEL1**	***Haemoproteus sp*.**	**JQ749720**	**1**	**-**
**CARDUEL2**	***Haemoproteus sp*.**	**JQ749721**	**1**	**-**
**CARDUEL3**	***Haemoproteus sp*.**	**JQ749722**	**1**	**-**
**SYMEL1**	***Haemoproteus sp*.**	**JQ749723**	**1**	**1**
**TURDUS3**	***Plasmodium sp*.**	**JQ749724**	**1**	**-**
PADOM01	*Plasmodium sp*.	DQ058611	2	5
PADOM02	*Plasmodium sp*.	DQ058612	1	15
PADOM05	*Haemoproteus passeris*	HM146898	2	8
PADOM08	*Plasmodium sp*.	GU065648	1	1
PADOM22	*Haemoproteus sp*.	GU065650	1	1
PAHIS1	*Haemoproteus sp*.	GU065651	1	4
SGS1	*Plasmodium relictum*	AF495571	9	123
RTSR1	*Plasmodium sp*.	AF495568	1	15
SYAT05	*Plasmodium vaughani*	DQ847271	2	28

Newly detected parasite lineages are marked in bold. In order to give a global vision we also include the number of host species infected in MalAvi database.

### Factors affecting prevalence

**Fig 1:**
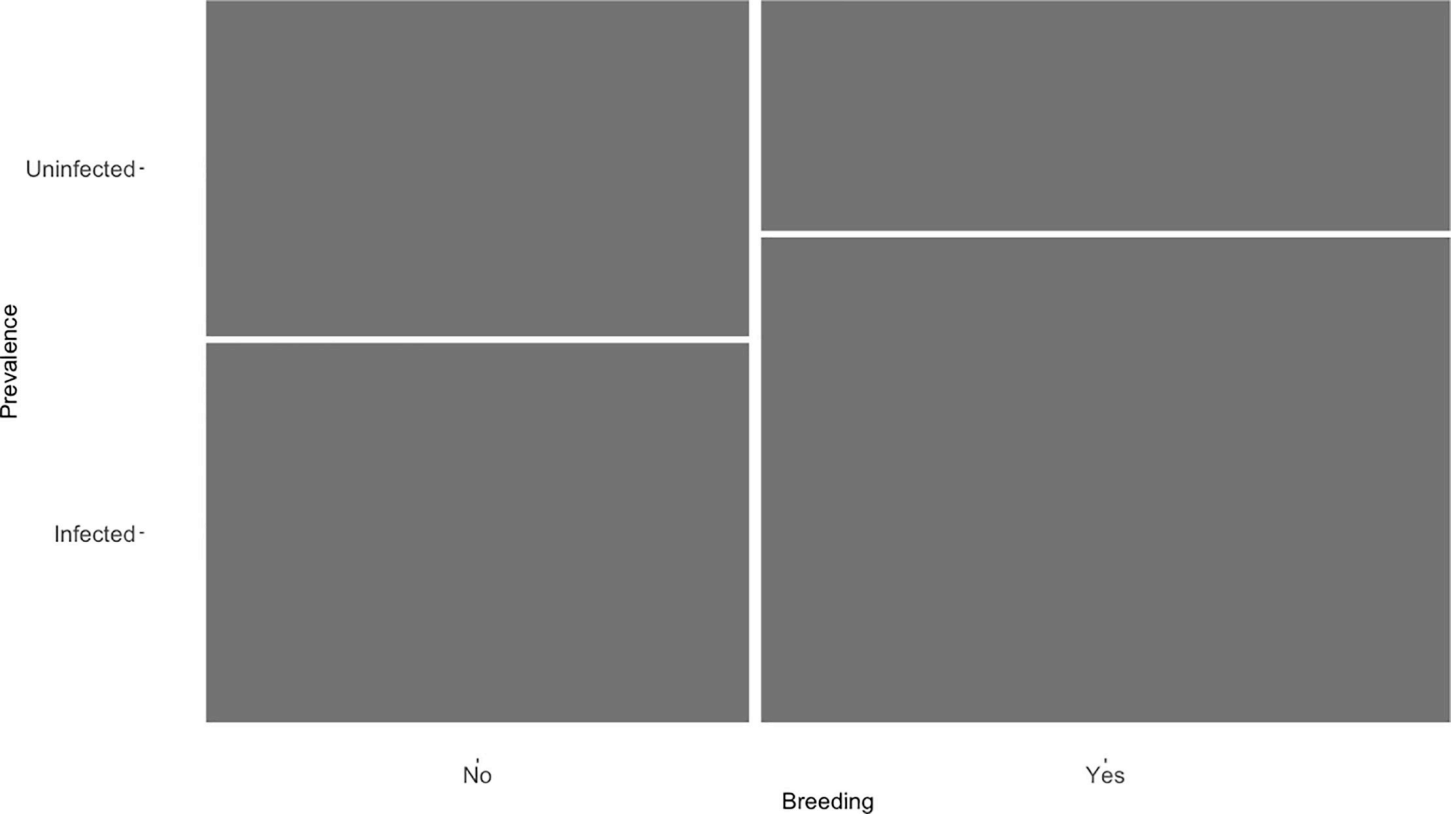
Proportion of individuals infected and uninfected during the breeding and non-breeding season.

Our statistical analyses suggest that parasite prevalence did not differ among the day of capture while the infection status was higher during breeding seasons (Table 3), i.e. one individual might have a higher probability of getting infected during the breeding season than during non-breeding season (Fig 1). Concerning random effects, interaction between parasite lineage and host species seemed to explain a high proportion of the variance in prevalence. Additionally, parasite lineage *per se* did seem to explain a proportion of the variance in parasite prevalence from the bird community while host bird species explain a low variation (Table 3). Finally, year and phylogeny of both bird and parasite did not explain an important variation of the dependent variable (Table 3).

**Table 3:** Results of both models: models 1 and 2.

	Bird (model 1)			Parasite (model 2)		
	Regression slope	Lower/upper 95% CI	*p*MCMC	Regression slope	Lower/upper 95% CI	*p*MCMC
*Fixed factors*						
Day	-0.121	-0.287/ 0.066	0.184	-0.184	-0.210/ 0.075	0.177
**Breeding season**	**2.258**	**0.271 / 4.624**	**0.052**	**2.112**	**0.159 / 4.783**	**0.049**
	Variance	Lower/upper 95% CI		Variance	Lower/upper 95% CI	
*Random factors*						
Year	0.054	0.016/0.238		0.061	0.017/0.247	
**Lineage**	**0.127**	**0.0001/0.241**		**0.105**	**0.0002/0.28**	
Individual	0.0001	0.00001/0.002		0.0001	0.00001/0.002	
Species	0.001	0.00002/0.054		0.0005	0.00001/0.074	
Linage*Species	**0.829**	**0.514/0.901**		**0.799**	**0.593/0.966**	
Phylo	0.002	0.00001/0.005		0.0004	0.00002/0.054	

Results from fixed factors are showing by the regression slope with the CI values 95%. Results from fixed factors are showed as the variation (Var) that each factor explain from the dependent variable. Values in bold represent factors that were statistically significant. Modeled with a logit link function.

### Infected bird species and phylogenetic distances

**Fig 2:**
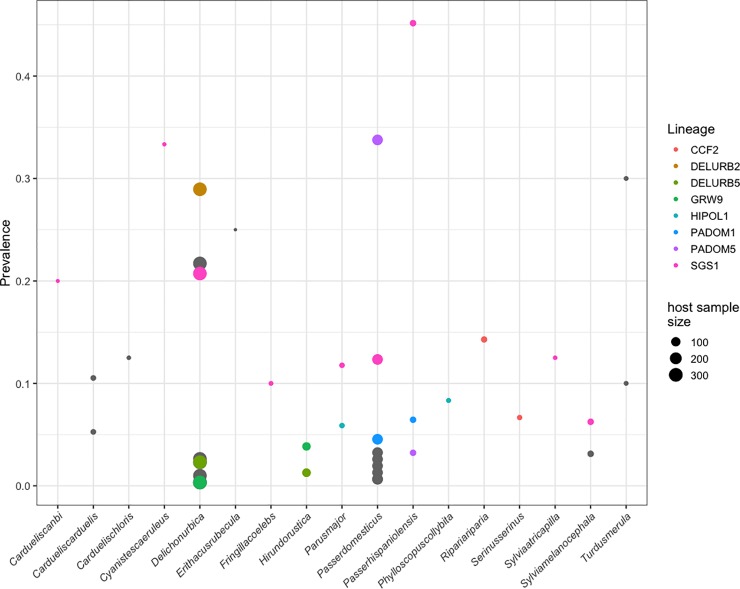
Prevalence of each parasite lineage in every host species found in the community. Bird species with no infection are not present in the figure. Lineages infecting more than one bird species have been colored marked while parasite lineages infected only one host species remain in grey. Note that depending on the underlying sample size, the size of the dots is proportional to the number of individuals.

Random effects for parasite lineage shows that there are considerable differences among parasite lineages in their host exploitation strategies. We found that the most prevalent parasite lineage was *Plasmodium relictum* SGS1 as it was detected in 9 out 21 host species. However, the prevalence in particular host species was quite variable (see Fig 2). In contrast, the other lineages seem to be more specialists, at the present study, as they were detected in fewer species.

In our community we found parasite lineages infecting one bird species (mean prevalence = 0.008 ± 0.02) and more than one bird species (mean prevalence = 0.04 ± 0.06). Within the latter, we detected 8 different lineages infecting, at least, two host species (Fig 2). Thus, we analysed the phylogenetic distance between the hosts species infected by one relative to the average phylogenetic distance between two species on the entire tree. These results allow us to differentiate between parasite lineages infecting close (mean prevalence = 0.03 ± 0.04) or distantly related (mean prevalence = 0.04 ± 0.06) bird species. Specifically, our results showed that *Haemoproteus* spp. CCF2 lineage, *Plasmodium* spp. HIPOL1 and *P*. *relictum* SGS1 infected distantly related bird species. Moreover, we detected that *Haemoproteus passeris* PADOM05 lineage, *Haemoproteus* spp. DELURB2 lineage, *Plasmodium* spp. DELURB5, GRW9 and PADOM01 lineages tended to infect closely related bird species (Table 4).

**Table 4:** Phylogenetic dispersion of parasite lineages infecting more than one host species.

	*N*	MPD_obs_	Mean ± SD	*Z*	*P*
SGS1 *P*. *relictum*	9	79.976	81.829 ± 2.609	-0.710	0.222
**PADOM05 *H*. *passeris***	**2**	**5.546**	**82.695±17.199**	**-4.485**	**0.002**
CCF2 *Haemoproteus* sp.	2	91.536	80.881±18.287	0.582	0.853
**DELURB2 *Haemoproteus* sp.**	**2**	**33.574**	**82.087±17.088**	**-2.838**	**0.038**
**DELURB5 *Plasmodium* sp.**	**2**	**33.574**	**82.111±17.851**	**-2.713**	**0.047**
**GRW9 *Plasmodium* sp.**	**2**	**33.574**	**81.479±18.615**	**-2.573**	**0.054**
HIPOL1 *Haemoproteus* sp.	2	86.983	81.817±17.714	0.291	0.304
**PADOM1 *Plasmodium* sp.**	**2**	**5.546**	**81.385±18.107**	**-4.188**	**0.003**

*N* = number of bird species infected. Values in bold represent parasite lineages that were detected in close phylogenetic bird species.

## Discussion

In this study of haemosporidian parasites infecting a community of wild birds breeding in the same area, we found that variation in infection status might be mainly affected by the interaction between host bird species and slightly by parasite lineage. We also found that during breeding season infection status is significantly higher than during non-breeding season. Additionally, we showed that *P*. *relictum* SGS1 is present in most of the bird species but with variable prevalence, while other parasites are more specialist parasites infecting fewer and close related host species as *H*. *passeris* PADOM05. Below, we discuss these main results in detail.

Virulence of *Plasmodium* and *Haemoproteus* spp. might be highly variable between species and lineages. Some malaria parasites might affect host fitness [65,66] and others might even influence survival [67–69]. Conversely, host bird species might also differ in the prevalence displayed due to the tolerance or susceptibility of each organism [16]. In this sense, the interaction between parasite lineage and host bird species ultimately determines infection severity and therefore prevalence. This idea has been previously suggested by Medeiros et al. [35] where they found that the host-pathogen compatibility might determine the parasite range. Our results support this hypothesis where variation in prevalence was mainly affected by the interaction between parasite lineage and bird host species. In this sense, it has been showed that avian malaria parasites (*Plasmodium ashfordi*) express a particular host-specific expression pattern depending on the infected bird [16]. That study highlighted the role that the interaction between host immune defence (susceptibility or resistance to parasites) and parasite strategy (virulence) may play in the prevalence displayed. Despite the number of studies focused on host-parasite interactions, the knowledge about the mechanisms behind it is still limited [70,71].

With regard to the previous results, variation in infection status was slightly affected by parasite lineage. These results emphasize the importance not only of the host-parasite interaction *per se* but also the parasite exploitation strategy that might vary depending on the bird identity [16]. In this sense, it has been shown that avian malaria parasites might develop a wide range of different strategies in order to avoid host defenses. For example, *Plasmodium* spp. can display some evasive mechanisms evading host defenses by clonal antigenic switches [72] or even avoid consequences of recognition by the immune system [73]. Additionally, it has been shown that, during mix infections, these parasite might compete with one another for access to the available hosts, thus, some lineages show more prevalence in certain bird species simply because an specific lineage may eliminate other parasite species [30]. Hence, parasite lineage might play an essential role determining the prevalence displayed in a host rather the host bird species itself.

According to our results, breeding season might affect the probability of becoming infected in the community. Previous studies have shown that, during the breeding season, prevalence of haemosporidian parasites can significantly increase [74,75] as a consequence of vector availability. The increase of vectors in the community may expand blood parasites among individuals and, therefore, increase prevalence [42]. In this sense, previous studies have suggested that the increase of vector availability might produce a proliferation of blood parasites during breeding season [76,77]. Our results agree with this idea pointing out a clear connexion between breeding season, vector availability and prevalence of blood parasites.

Although the RBH and TOH hypotheses have been broadly discussed (e.g. [38,78]) there is still no clear consensus about the importance of AOR within parasite ecology research. In this sense, and despite a considerable number of studies supporting the idea that specialist parasites might show higher prevalence than generalist parasites [79], some studies have found different result supporting RBH. For instance, in Hawaii, birds *Plasmodium relictum* caused the extinction of native species of honeycreepers [80,81] despite being one of the most generalist parasite species. In this sense, it has been suggested that generalist parasites (*P*. *relictum* GRW4 and SGS1) have the ability to infect a broad range of host species, but also display a high prevalence in some of them [82]. Our results agree with this hypothesis, where *P*. *relictum* SGS1 was detected in most of the bird species infected, and it displayed differences in prevalence depending on the bird species. Moreover, this haemosporidian parasite lineage was detected in unrelated host species highlighting the parasite’s ability to adapt to distantly related host species and emphasizing the parasite’s generalist strategy. In the case of *P*. *relictum* SGS1 the number of potential host species is 123 [20], where every host individual and species provide a different environment that need to be overcome by the plasticity of parasites [83]. Although the mechanism underlying this plasticity is still unknown, it has been recently shown that the reticulocyte binding proteins, a polymorphic gene family involved in host cell invasion and attributed to host specificity [71], is significantly expanded in *P*. *relictum* [84]. This discovery could explain the ability of this parasite species to infect a wide range of avian species and its overwhelming capacity to infect a wide range of host species [84]. Nevertheless, further studies are needed in order to assess mechanisms allowing generalist parasites to be present in a large number of bird species.

Finally, our analysis revealed that the number of infected bird species by the same parasite lineage does not determine whether a parasite may be more or less specialist. In this sense, *Haemoproteus* spp. CCF2, HIPOL1 lineages were detected in distantly related bird species suggesting that, although each of these lineages was only detected in two bird species, they are not strict specialist parasites [38]. Additionally, *Haemoproteus* spp. PADOM05, DELURB2 lineages and *Plasmodium* spp. DELURB5, PADOM1, GRW9 spp. lineages were detected in closely related bird species suggesting that these lineages may tent to be specialist parasites [38] in our community. The prevalence displayed by these parasite lineages varied depending on the bird species infecting. Thus, in house sparrows (*Passer domesticus*) prevalence was higher for specialist parasites (*Haemoproteus* spp. PADOM05) than for generalist parasites. In contrast, Spanish house sparrows (*Passer hispaniolensis*) displayed prevalence was higher for generalist parasites (*P*. *relictum* SGS1). These results agree with previous studies suggesting that, in certain cases, specialist parasites might arise higher prevalence than generalist parasites [79] but, in other cases, generalist manage to reach high prevalence [82]. This could be because generalist parasites can be better adapted to a subset of host species where they will present higher infection status [85]. Our results can only confirm that the association of the parasite lineage and host bird species affects prevalence, and that this association is the ultimate factor affecting the prevalence in the community studied.

In conclusion, we have shown that, at the community level, the interaction between host and parasite identity might have a stronger effect on the variation of infection status than bird host species or parasite lineage alone. These results highlight the importance of studies focusing on the community level and analysing how different parasite lineages might interact with a variety of host species. The present study also emphasizes that even parasites (such *as P*. *relictum* SGS1 and *H*. *passeris* PADOM05) that are considered as generalist or a specialist can display both high and low prevalence per host species. Taken as a whole, these results suggest that detected prevalence mainly result from the interaction between host immune defences and parasite exploitation strategy where parasite lineage might play an important role following an approach depending on the bird species.

## Supporting information

S1 Fig Phylogenetic associations between *Plasmodium* and *Haemoproteus* parasites and their avian hosts. (TIFF)

S1 Table Pairwise distance matrix within new parasite lineages detected in the present study (in bold) and close related parasite lineages according to parasite phylogenetic tree (S1 Fig). (DOCX)

S1 Data Database used for all the statistical analyses. Plots were also based on this database.
(ZIP)
