# Supplementary figures and images for: Host-parasite interaction explains variation in the prevalence of avian haemosporidians at the community level
Source: PLoS One. 2019 Mar 6;14(3):e0205624. doi: 10.1371/journal.pone.0205624 (PMC6402683; doi:10.1371/journal.pone.0205624)

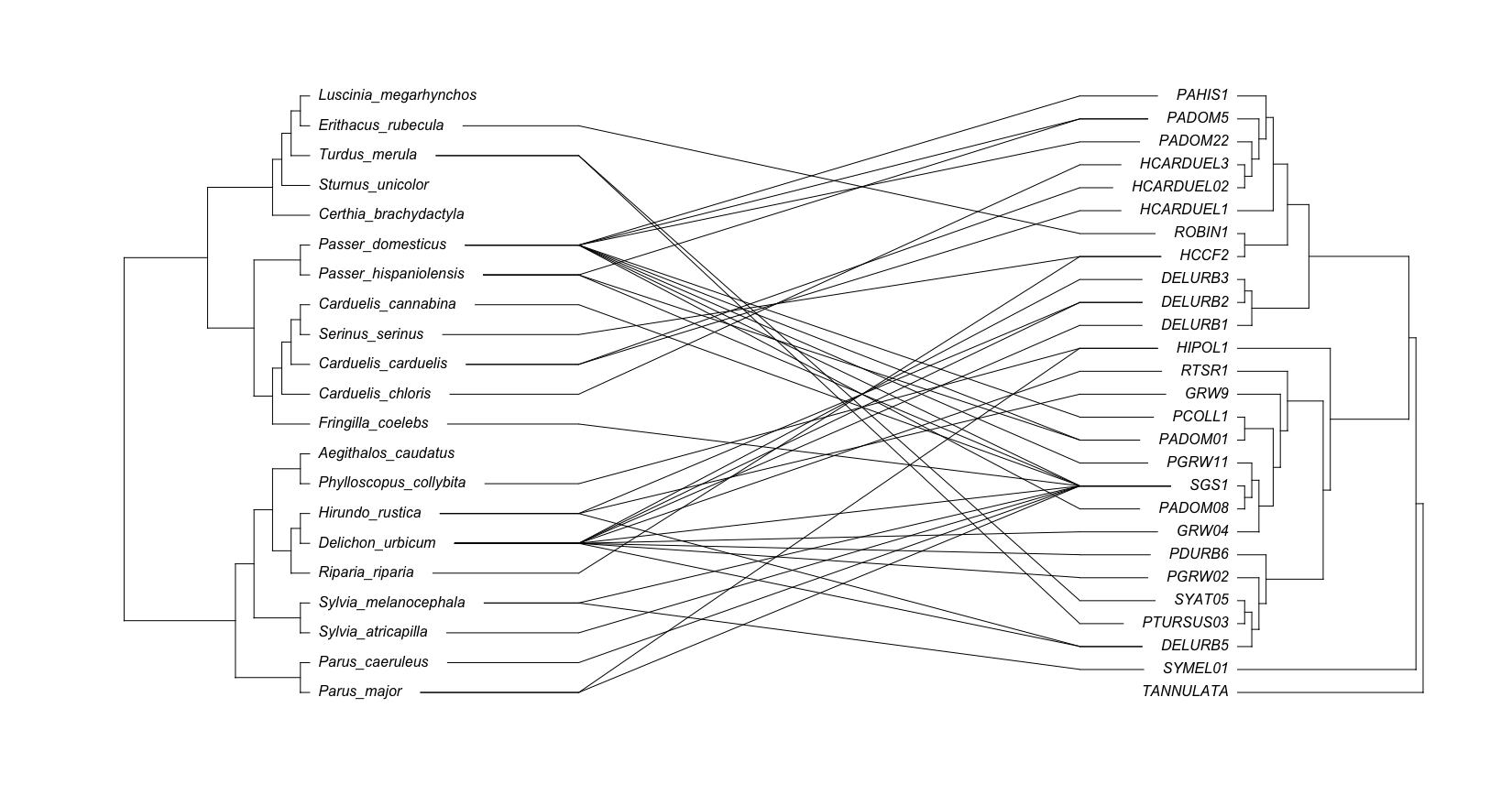

Supplement: S1 Fig — (TIFF) [file pone.0205624.s001.tiff]
