# Supplementary material for: Host-parasite interaction explains variation in the prevalence of avian haemosporidians at the community level
Source: PLoS One. 2019 Mar 6;14(3):e0205624. doi: 10.1371/journal.pone.0205624 (PMC6402683; doi:10.1371/journal.pone.0205624)
Supplement: S1 Table — (DOCX) [file pone.0205624.s002.docx]

|  | 1 | 2 | 3 | 4 | **5** | **6** | **7** | 8 | 9 | 10 | **11** | 12 | 13 |
| --- | --- | --- | --- | --- | --- | --- | --- | --- | --- | --- | --- | --- | --- |
| 1. DELURB1 |  |  |  |  |  |  |  |  |  |  |  |  |  |
| 2. DELURB2 | 0.019 |  |  |  |  |  |  |  |  |  |  |  |  |
| 3. DELURB3 | 0.019 | 0.005 |  |  |  |  |  |  |  |  |  |  |  |
| 4.DELLURB5 | 0.125 | 0.0120 | 0.123 |  |  |  |  |  |  |  |  |  |  |
| **5. CARDUELIS1** | **0.076** | **0.071** | **0.071** | **0.117** |  |  |  |  |  |  |  |  |  |
| **6. CARDUELIS2** | **0.073** | **0.068** | **0.068** | **0.117** | **0.024** |  |  |  |  |  |  |  |  |
| **7. CARDUELIS3** | **0.073** | **0.068** | **0.068** | **0.117** | **0.019** | **0.005** |  |  |  |  |  |  |  |
| 8. CCF2 | 0.068 | 0.068 | 0.071 | 0.131 | **0.076** | **0.063** | **0.063** |  |  |  |  |  |  |
| 9. PADOM05 | 0.078 | 0.071 | 0.073 | 0.107 | **0.024** | **0.014** | **0.009** | 0.063 |  |  |  |  |  |
| 10. DURB06 | 0.133 | 0.123 | 0.125 | 0.091 | **0.123** | **0.125** | **0.120** | 0.120 | 0.120 |  |  |  |  |
| **11. TURDUS3** | **0.131** | **0.128** | **0.128** | **0.061** | **0.123** | **0.117** | **0.117** | **0.131** | **0.123** | **0.083** |  |  |  |
| 12. ROBIN1 | 0.076 | 0.076 | 0.076 | 0.128 | **0.038** | **0.033** | **0.031** | 0.073 | 0.036 | 0.133 | **0.133** |  |  |
| **13. SYMEL01** | **0.081** | **0.076** | **0.078** | **0.101** | **0.046** | **0.048** | **0.076** | **0.076** | **0.043** | **0.120** | **0.101** | **0.051** |  |

**S1 Table:** Pairwise distance matrix within new parasite lineages detected in the present study (in bold) and close related parasite lineages according to parasite phylogenetic tree (S1 Fig).
